# Microneedling for Cutaneous Manifestations of Autoimmune Disease: A Scoping Review

**DOI:** 10.7759/cureus.107648

**Published:** 2026-04-24

**Authors:** Julia Juriga, Erica K Rankin, Melanie Rodriguez, Nabiha T Atiquzzaman, Amanda Morello, Claudia Pedreira, Matthew T McKinley, Kiarra Krulikowski, Dania Ilyas, Daniela Prado Escobar, Lily Tehrani, Shane Williams, Robin J Jacobs

**Affiliations:** 1 Medicine, Nova Southeastern University Dr. Kiran C. Patel College of Osteopathic Medicine, Fort Lauderdale, USA

**Keywords:** alopecia areata, autoimmune disease, cutaneous manifestations, dermatology, dermatomyositis, microneedling, transdermal drug delivery, vitiligo

## Abstract

Microneedling has emerged as a promising, minimally invasive modality for treating cutaneous manifestations of autoimmune diseases, including alopecia areata, vitiligo, and dermatomyositis. This procedure induces controlled microinjuries that stimulate wound healing, collagen production, and tissue remodeling, while also enhancing transdermal drug delivery. This scoping review aimed to evaluate the current evidence on the efficacy of microneedling in managing autoimmune-related dermatologic conditions. The review was conducted in accordance with the PRISMA-ScR guidelines. Peer-reviewed studies were identified through Medical Literature Analysis and Retrieval System Online (MEDLINE), Cumulative Index to Nursing and Allied Health Literature (CINAHL), and Web of Science, a multidisciplinary citation database of scientific literature. Eligible studies were published in English between 2014 and 2024, involved adult populations, and reported original research. Review articles, non-human studies, and studies involving participants under 18 years of age were excluded. Fourteen studies met the inclusion criteria. Overall, microneedling demonstrated benefit primarily as an adjunctive therapy, with improved outcomes reported when combined with treatments such as corticosteroids, platelet-rich plasma, and narrowband ultraviolet B therapy. Evidence supports its role in promoting hair regrowth in alopecia areata and facilitating repigmentation in vitiligo. Limited evidence from a case report suggests potential benefit in reducing calcinosis cutis in dermatomyositis. The therapeutic effects of microneedling appear to be mediated by enhanced drug absorption and stimulation of local regenerative pathways. Although findings are promising, the current evidence is limited by small sample sizes, heterogeneity in study design, and short follow-up periods. Larger, well-designed randomized controlled trials are needed to establish standardized treatment protocols and to better assess long-term safety and efficacy. Microneedling represents a potential adjunctive treatment option for autoimmune-related cutaneous conditions and warrants further investigation.

## Introduction and background

Autoimmune diseases are characterized by a breakdown in immunological tolerance, in which B and T lymphocytes fail to recognize self-antigens, resulting in immune-mediated destruction of healthy tissues [1]. The global incidence of autoimmune diseases continues to rise, underscoring the need for improved prevention and treatment strategies [2]. Autoimmunity may be classified as physiological, in the absence of clinical disease, or pathological, in which loss of tolerance leads to inflammation and tissue damage driven by self-reactive lymphocytes [3]. Many autoimmune conditions, including lupus erythematosus, dermatomyositis, and scleroderma, present with cutaneous manifestations such as papulosquamous plaques, pigmentary changes, pruritus, and lichenification, all of which can significantly impair quality of life [4]. Standard management typically includes topical and systemic corticosteroids or immunosuppressive therapies [4]. However, these treatments are often associated with adverse effects, including hematologic toxicity, increased susceptibility to infection, and elevated malignancy risk, prompting interest in alternative therapeutic approaches [5].

The concept of harnessing the body’s intrinsic regenerative capacity has led to growing interest in minimally invasive techniques such as microneedling. This procedure involves using fine needles to create controlled microinjuries in the skin, thereby stimulating wound healing, collagen synthesis, and tissue remodeling. The resulting microchannels promote dermal vasodilation, keratinocyte migration, and stem cell activation, facilitating the release of growth factors essential for neovascularization and neocollagenesis [6]. Originally inspired by acupuncture, microneedling has evolved into a widely utilized modality across both therapeutic and cosmetic dermatologies [7].

Several microneedling devices are currently available, including dermarollers and automated pen-like devices such as the Dermapen. Dermarollers consist of medical-grade rollers embedded with multiple fine needles, while automated devices allow for adjustable needle depth and controlled, stamp-like application to create uniform microchannels [8,9]. The procedure is generally well tolerated and relatively quick, although multiple treatment sessions are typically required. Pretreatment with topical agents, such as vitamins A and C, is often recommended to enhance collagen induction, and appropriate patient counseling is essential to set expectations regarding gradual clinical improvement [10].

Microneedling has demonstrated efficacy in a wide range of dermatologic conditions, including scarring, inflammatory dermatoses, photoaging, and hair loss disorders [11-14]. It is frequently used in combination with adjunctive therapies, such as topical corticosteroids, minoxidil, platelet-rich plasma, laser therapy, and radiofrequency, to enhance therapeutic outcomes [12,14]. In addition to its direct effects on tissue remodeling, microneedling functions as a transdermal drug delivery system by temporarily disrupting the skin barrier, thereby increasing local drug penetration while minimizing systemic exposure [15,16]. Histologic studies have demonstrated substantial increases in collagen and elastin deposition, as well as increased epidermal thickness following treatment [15].

Emerging research has explored the application of microneedling in immune-mediated dermatologic conditions and transdermal immunomodulation [17-19]. For example, microneedle-based systems have been investigated for the delivery of regulatory T cells in psoriasis to enhance anti-inflammatory responses [17]. Despite its therapeutic potential, safety remains an important consideration. Microneedling induces a localized inflammatory response and may facilitate microbial entry into deeper skin layers, particularly if not performed under appropriate sterile conditions [20]. Reported adverse effects are generally mild and include transient pain, erythema, hyperpigmentation, and superficial infection [21,22]. The balance between therapeutic benefit and potential risk is particularly relevant in autoimmune populations, where disease exacerbation remains a theoretical concern.

Interest in microneedling continues to grow, driven by increasing patient demand for non-surgical treatment options and its applicability across diverse skin phototypes [23,24]. Advances in device fabrication have further improved accessibility and cost-effectiveness, supporting broader clinical adoption [25]. Despite this growing interest, the role of microneedling in autoimmune dermatologic disease remains incompletely characterized. Existing studies suggest potential benefits across conditions such as alopecia areata, vitiligo, lupus, and dermatomyositis; however, the evidence is heterogeneous and has not been comprehensively synthesized [14].

Accordingly, this scoping review aimed to evaluate the extent and nature of the current literature on microneedling for the management of cutaneous manifestations in adults with autoimmune diseases. The primary research question guiding this review was: *What is the effect of microneedling on cutaneous manifestations in adults with alopecia areata, vitiligo, lupus, and dermatomyositis?*

## Review

Methods

Eligibility Criteria

Studies were included if they met the following criteria: (1) peer-reviewed original research, (2) published in English between 2014 and 2024, and (3) involved adult populations (≥18 years). Review articles, non-human studies, and studies including participants younger than 18 years were excluded. These criteria were selected to ensure inclusion of contemporary, primary evidence relevant to adult populations and current clinical practice. The review was conducted in accordance with the Preferred Reporting Items for Systematic Reviews and Meta-Analyses extension for Scoping Reviews (PRISMA-ScR) guidelines.

Information Sources

A comprehensive literature search was conducted using Medical Literature Analysis and Retrieval System Online (MEDLINE), Cumulative Index to Nursing and Allied Health Literature (CINAHL), and Web of Science, a multidisciplinary citation database of scientific literature. Searches were performed between September and October 2024, and search strategies were adapted for each database. Both experimental and quasi-experimental study designs were considered. A preliminary search confirmed that no existing systematic or scoping reviews addressing this topic were available. This review followed the Joanna Briggs Institute (JBI) methodology for scoping reviews.

Search Strategy

Searches were conducted using combinations of the following keywords: microneedling, autoimmune, vitiligo, dermatomyositis, lupus, and alopecia. Boolean operators (AND/OR) were applied as appropriate for each database. The initial search yielded 333 articles from CINAHL (n = 27), Web of Science (n = 149), and MEDLINE (n = 157). Table 1 reports the search strategies by database. After the removal of 157 duplicate records, the remaining 176 reports were screened for eligibility.

**Table 1:** Database search strategy MEDLINE: Medical Literature Analysis and Retrieval System Online, CINAHL: Cumulative Index to Nursing and Allied Health Literature, Web of Science: multidisciplinary citation database of scientific literature.

Database	Keywords searched	Number of articles
CINAHL (09/09/2024 )		
#1	“Microneedling” AND “dermatology”	12
#2	“Microneedling” AND “Autoimmune diseases”	3
#3	#1 OR #2	15
#4	“Microneedling”	158
#5	“dermatomyositis” OR “lupus” OR “vitiligo” OR “alopecia”	25,484
#6	#4 AND #5	27
Web of Science (09/09/2024)		
#1	“Microneedling” AND “autoimmune”	10
#2	“Microneedling”	712
#3	“dermatomyositis” OR “lupus” OR “vitiligo” OR “alopecia”	170,128
#4	#2 AND #3	149
MEDLINE (10/01/2024)		
#1	“dermatomyositis” OR “lupus” OR “vitiligo” OR “alopecia”	156,628
#2	“Microneedling”	709
#3	“Microneedling” AND “autoimmune”	10
#4	#2 AND #1	157

Study Selection

**Figure 1:**
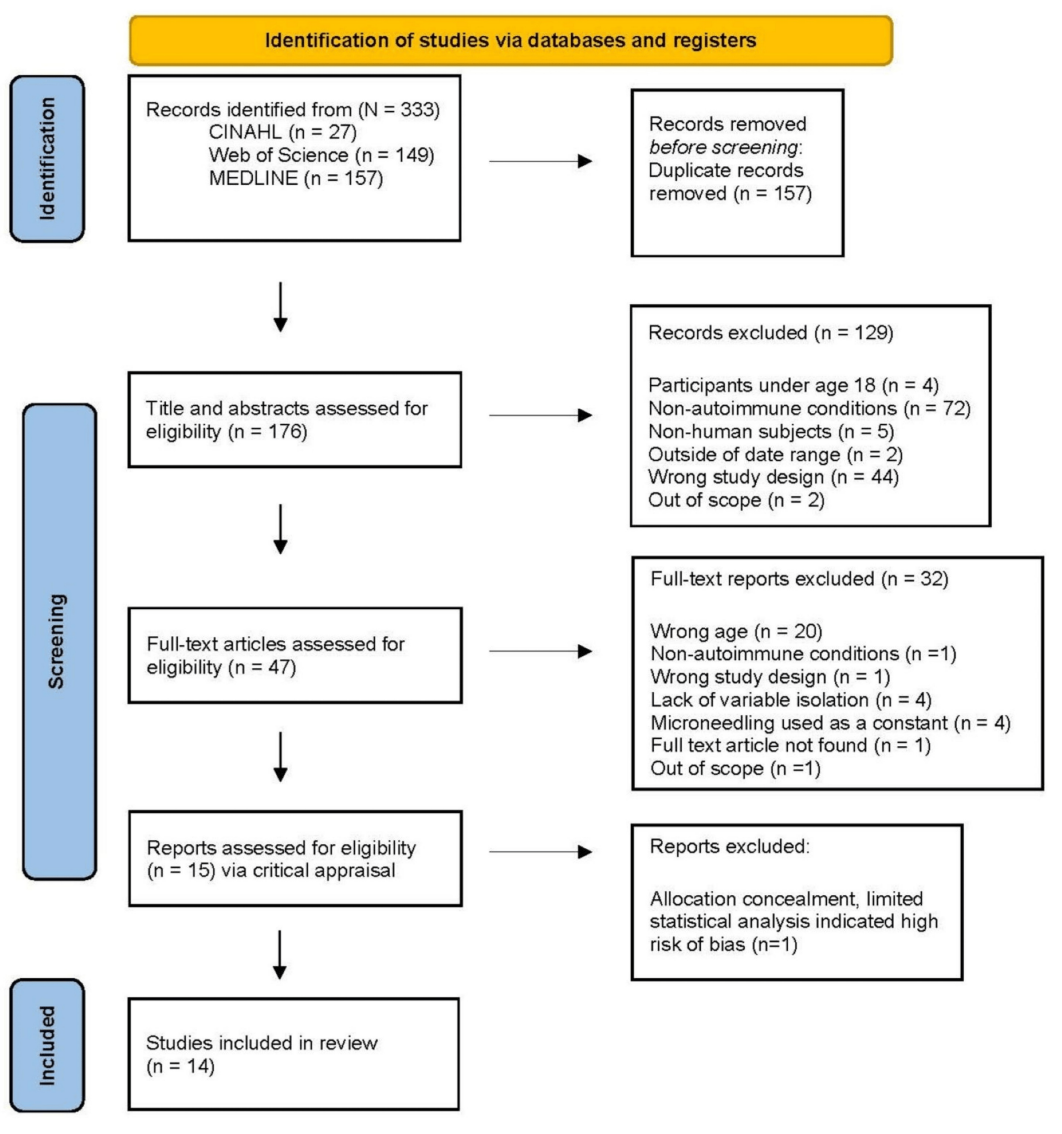
PRISMA flow diagram of the search and selection process PRISMA: Preferred Reporting Items for Systematic Reviews and Meta-Analyses

Two reviewers independently screened the 176 titles and abstracts for relevance based on the predefined inclusion criteria; 129 records were excluded. Articles meeting initial screening criteria underwent full-text review by two independent reviewers (n = 47). Discrepancies at both stages were resolved through discussion and consensus, with involvement of a third reviewer when necessary. The study selection process was documented using a PRISMA-ScR flow diagram (Figure 1).

Data Charting Process

A standardized data extraction form was developed using Microsoft Excel (Microsoft Corp., Redmond, WA, USA). Two reviewers independently extracted data from included studies, including study characteristics (e.g., population, intervention, design), outcomes, and key findings relevant to the review question. The data extraction process was iteratively refined to ensure consistency and accuracy. Data management was supported using Google-based platforms (Google, Mountain View, CA, USA) and Rayyan (Rayyan Systems Inc., Cambridge, MA, USA), a web-based platform designed to help researchers screen and organize studies for systematic reviews, scoping reviews, and meta-analyses.

Critical Appraisal of Sources of Evidence

Critical appraisal of included studies was conducted to ensure the validity and methodological rigor of the evidence synthesized in this scoping review. Following initial (tier-one) screening, all potentially eligible articles underwent detailed quality assessment using the Joanna Briggs Institute (JBI) Critical Appraisal Tools. These tools provide a structured approach for evaluating study quality, including assessment of bias, methodological congruence, and key elements of study design [26]. 

Studies were categorized as low (>70%), moderate (50%-70%), or high (<50%) risk of bias based on appraisal scores. Sixteen articles underwent independent, blinded critical appraisal by two reviewers. Discrepancies were resolved through discussion and consensus. Following quality assessment, 14 studies met the inclusion criteria and were classified as low risk of bias. This process helped ensure that only methodologically robust studies were included in the final synthesis. Only studies meeting predefined methodological quality thresholds were included in the final synthesis.

Synthesis of Results

Included studies were grouped according to the type of autoimmune dermatologic condition (e.g., alopecia areata, vitiligo, dermatomyositis). Findings were synthesized descriptively, including study design, population characteristics, interventions, and key outcomes, and presented in tabular format.

Results 

Following a systematic screening process, 14 studies met the inclusion criteria and were included in this scoping review, all of which explored the use of microneedling for cutaneous manifestations of autoimmune conditions, including alopecia areata, vitiligo, and dermatomyositis. These studies assessed microneedling either as a standalone therapy or in combination with treatments such as topical corticosteroids, tacrolimus, platelet-rich plasma (PRP), and narrowband ultraviolet B (NB-UVB) therapy. Although studies related to lupus were identified during the search process, none met the predefined inclusion criteria and were therefore excluded from the final analysis. Overall, microneedling enhanced drug absorption, promoted hair regrowth in alopecia areata, and facilitated repigmentation in vitiligo. Across the included studies, the most frequently reported starting needle depth was 0.5 mm, with several studies subsequently increasing depth to 1.0-1.5 mm depending on treatment response and device type. Several randomized controlled trials found that combination therapies were more effective than monotherapy.

Alopecia Areata

Six studies assessed the role of microneedling in alopecia areata. Of the studies, two were case reports, one retrospective study, and three clinical trials. These studies included 360 subjects, of which microneedling was utilized in 290 patients. Overall, microneedling was found to improve hair regrowth in patients with alopecia areata. Microneedling was used as adjunct therapy in all studies, most commonly as a vehicle for improved topical cream penetration.

Vitiligo

Vitiligo was the most common dermatologic condition assessed, with eight studies directed towards microneedling utilization. Sample sizes of these studies were much larger, and a total of 329 patients with vitiligo were included. Study designs included four randomized controlled trials, three comparative studies, and one clinical trial. As with alopecia areata, microneedling was used as an adjunct therapy in vitiligo treatment. Common adjunct therapies included topical tacrolimus, pimecrolimus, latanoprost, and NB-UVB. When used in combination with topical medication, microneedling was found to increase pigmentation across all patients with vitiligo. Combination therapy NB-UVB and microneedling for repigmentation found no difference when compared to repigmentation with NB-UVB alone. However, NB-UVB and microneedling combination therapy was found to be more effective than topical steroids alone in the repigmentation of non-segmental vitiligo. Despite this, PRP was found to be more efficacious when compared to NB-UVB and microneedling combination therapy. These findings suggest microneedling benefits may vary in patients with vitiligo, emphasizing the need for more controlled clinical trials.

Dermatomyositis/Calcinosis Cutis

A single case report assessed the role of microneedling in dermatomyositis-associated calcinosis cutis, a condition characterized by calcium deposition in the skin and soft tissues [27]. Inkless tattooing over the affected lesions was associated with a reduction in calcification volume. This finding provides mechanistic insight into the potential benefit of microneedling and suggests a possible therapeutic role in dermatomyositis [28].

Efficacy and Safety

Microneedling was generally more effective when combined with other therapies than as a monotherapy. However, the studies reviewed had several limitations, including small sample sizes, short study durations, and inadequate assessment of patient discomfort and pain. Additionally, some studies lacked control groups, reducing statistical power and limiting generalizability. Future research should focus on evaluating the long-term effects, durability of outcomes, pain tolerability, and overall safety of microneedling for these conditions. Since vitiligo patients showed inconsistent responses, further studies are needed to clarify the effects of microneedling therapy in its management. A summary of the studies included in this review is reported in Table 2.

**Table 2:** Summary of included studies MN: microneedling, TA: triamcinolone acetonide, AA: alopecia areata, NB-UVB: narrowband ultraviolet B, PRP: platelet-rich plasma, FCL: fractional carbon dioxide laser, MMP®: microinfusion of medications into the skin, RCT: randomized controlled trial.

Reference	Country	Aim of the study	Sample size	Study design	Condition	Findings	Limitations
Motlaghzadeh et al. (2022) [28]	USA	Inkless tattooing	1 patient	Case report	Dermatomyositis	Reduced calcinosis	Single case
Esmat et al. (2021) [29]	Egypt	Enhance tacrolimus absorption	20 patients	RCT	Vitiligo	Improved absorption	Small sample
Ebrahim et al. (2021) [30]	Egypt	MN + tacrolimus vs tacrolimus	48 patients	RCT	Vitiligo	Combination superior	Small sample
Khalil et al. (2023) [31]	Egypt	PRP vs MN	30 patients	Comparative	Vitiligo	PRP superior	Small sample
Wei et al. (2024) [32]	China	MN + minoxidil/TA	230 patients	Clinical trial	Alopecia areata	Improved regrowth	Short follow-up
Arora et al. (2022) [33]	India	Compared MN to intralesional TA	60 patients	Prospective randomized trial	Alopecia areata	Improved drug delivery and hair regrowth	Small sample; short follow-up
Asad et al. (2020) [34]	USA	Treatment of ophiasis with MN	1 patient	Case report	Alopecia areata	Near-complete regrowth	Single case
Barletta et al. (2020) [35]	Brazil	MN + TA via MMP®	2 patients	Case report	Alopecia areata	Significant regrowth	No control
El-Magid et al. (2023) [36]	Egypt	MN + latanoprost	72 patients	Clinical trial	Vitiligo	~50% repigmentation	Short duration
Iraji et al. (2021) [37]	Iran	MN + pimecrolimus	15 patients	RCT	Vitiligo	Higher repigmentation	Small sample
Kaur et al. (2023) [38]	India	NB-UVB ± MN	30 patients	Comparative	Vitiligo	No added benefit	Short duration
Kiran et al. (2024) [39]	Pakistan	Tacrolimus ± MN	30 patients	Comparative	Vitiligo	Combination effective	Short duration
Mansouri et al. (2024) [40]	Iran	FCL vs MN combos	84 patients	RCT	Vitiligo	MN + NB-UVB best	Blinding issues
Ragab et al. (2020) [41]	Egypt	PRP delivery methods	60 patients	RCT	Alopecia areata	MN + PRP improved outcomes	No control

As a scoping review, this study was not designed to perform quantitative synthesis or meta-analysis, and findings were summarized descriptively in accordance with the PRISMA-ScR guidelines.

Discussion

This scoping review highlights the potential benefit of microneedling as a supportive treatment for cutaneous manifestations of autoimmune diseases, including alopecia areata, vitiligo, and dermatomyositis. Across studies, microneedling was rarely used as monotherapy and demonstrated the greatest benefit when combined with established treatments. Its primary role appears to be enhancement of transdermal drug delivery and stimulation of local wound-healing pathways, including collagen production and growth factor release [6,16]. As a scoping review, this study was not designed to assess pooled effect sizes or establish causality.

Microneedling was primarily used as an adjunctive therapy in alopecia areata and vitiligo. Common successful co-therapies included topical nonsteroid creams, PRP, and NB-UVB therapy. In alopecia areata, several clinical trials and case-based studies reported improved hair regrowth when microneedling was used alongside PRP, triamcinolone acetonide, or topical minoxidil [14]. The proposed mechanism involves the creation of epidermal microchannels that facilitate deeper penetration of topical agents, in addition to the induction of localized growth factors that may promote transition into the anagen phase [6,16]. While short-term outcomes were favorable, most studies were limited by modest sample sizes and relatively brief follow-up periods, making it difficult to determine the long-term sustainability of regrowth or relapse rates.

Outcomes in vitiligo were heterogeneous. Several randomized and comparative studies demonstrated greater repigmentation when microneedling was combined with topical tacrolimus, pimecrolimus, corticosteroids, or latanoprost compared to topical therapy alone [14-16]. These findings suggest that the benefit may largely stem from improved drug absorption rather than a direct immunologic effect. In contrast, studies evaluating microneedling in combination with NB-UVB therapy showed inconsistent results, with some reporting no added benefit over NB-UVB alone. Additionally, PRP appeared superior to microneedling in certain comparisons. Variability in lesion stability, treatment duration, and procedural technique may explain these differences.

Dermatomyositis was the only condition that did not involve additional therapies; however, the only study assessing this condition used a nontraditional microneedling technique involving inkless tattooing. Findings from this study suggest that microneedling may serve as a potential treatment option for calcinosis cutis affecting patients with dermatomyositis; however, more studies are needed to better understand its therapeutic role. Because dystrophic calcinosis cutis is associated with impaired tissue repair and chronic inflammation, microneedling may help restore local tissue homeostasis and facilitate remodeling around calcium deposits [27]. More extensive treatment was followed by broader regression of calcifications, raising the possibility that both local tissue remodeling and systemic release of repair mediators contribute to the response [28].

Overall, the findings suggest that microneedling may be beneficial in select autoimmune dermatologic conditions; however, further studies are needed to better evaluate its efficacy as a standalone therapy. Additionally, microneedling has yet to be assessed in all autoimmune conditions. Adverse effects reported across studies were generally mild and transient, including procedural discomfort, erythema, post-inflammatory hyperpigmentation, and occasional superficial infection [21,22]. Although microneedling intentionally induces localized inflammation, there was no clear evidence of autoimmune disease exacerbation in the included studies. However, most trials were underpowered to detect rare or delayed complications, and long-term safety data remain limited. Findings should be interpreted within the context of predominantly small-scale and heterogeneous study designs.

Limitations

While the findings of this review are promising, several limitations should be acknowledged. Many of the studies included small sample sizes, short treatment courses, and limited blinding, restricting generalizability [29]. The included studies were heterogeneous in design, with some lacking control groups or randomization, potentially increasing the risk of bias. Outcome measures were inconsistent across studies, limiting the ability to compare due to variations in needle depth, frequency of sessions, and use of combination therapies [30,31]. Finally, limitations related to the predefined inclusion criteria may have excluded older, yet still relevant, research. Microneedling use has yet to be comprehensively assessed in existing autoimmune disorders with dermatologic relevance, as exemplified by the lack of results for lupus within our inclusion criteria. Despite clinical relevance, a lack of eligible human studies remains. This gap in the literature highlights an important area for future research. Variations in database indexing raise the possibility of the unintentional exclusion of relevant studies. Variability across studies in treatment protocols, needle depth, adjunctive therapies, and outcome measures limited direct comparability and highlights the need for standardized approaches in future research. As a scoping review, this study aimed to summarize and map the existing literature rather than perform quantitative synthesis.

Implications for Clinical Practice

Microneedling may offer promise as an adjunctive therapy in the treatment of autoimmune-related dermatologic conditions, especially in patients who are unable to tolerate systemic immunosuppressants [10,21]. Its potential for fewer side effects in comparison to traditional treatments makes it an attractive option. The majority of studies initiated treatment at a 0.5 mm needle depth, suggesting that this depth is commonly considered an effective and well-tolerated starting point for microneedling interventions. However, before routine clinical use can be recommended, standardized treatment protocols such as optimal number of treatment sessions, time intervals between treatments, and needle depth must be established in order to ensure patient safety and efficacy tailored to each specific autoimmune condition [22].

Implications for Future Research

Future large-scale randomized controlled trials with longer follow-up periods are needed to establish microneedling protocols for the management of autoimmune skin conditions [32]. Patient-reported outcomes, particularly those related to quality of life, should be examined to better assess the broader impact of microneedling in dermatological care [10]. Further investigation into the interaction between microneedling and the immune system may provide greater insight into the procedure's mechanism of action [28]. Exploring the benefit of microneedling in conjunction with biologic therapies may introduce alternative treatment interventions for dermatologic manifestations of autoimmune disorders [17].

## Conclusions

Microneedling, as a minimally invasive intervention, demonstrated promising outcomes for skin-related manifestations of autoimmune diseases. While initial studies suggest benefit in conditions such as alopecia areata, vitiligo, and dermatomyositis, further research is necessary to determine its long-term safety and efficacy. Larger randomized controlled trials and the development of standardized protocols are essential to ensure the safe and effective incorporation of microneedling into clinical practice. Future studies should focus on evaluating long-term outcomes and refining treatment parameters to establish microneedling as a reliable adjunctive therapy for autoimmune-related cutaneous conditions.
